# Seroprevalence of bovine viral diarrhea virus in crossbred dairy cattle in Bangladesh

**DOI:** 10.14202/vetworld.2017.906-913

**Published:** 2017-08-12

**Authors:** Mohammed Arif Uddin, A. S. M. Lutful Ahasan, Kamrul Islam, Md. Zohorul Islam, Altaf Mahmood, Ariful Islam, Kazi Muhammad Fakhrul Islam, Abdul Ahad

**Affiliations:** 1 Department of Livestock Services, Ministry of Fisheries and Livestock, Government of the People’s Republic of Bangladesh, Bangladesh; 2 Department of Microbiology and Veterinary Public Health, Faculty of Veterinary Medicine, Chittagong Veterinary and Animal Sciences University, Bangladesh; 3 Department of Anatomy and Histology, Faculty of Veterinary Medicine, Chittagong Veterinary and Animal Sciences University, Bangladesh; 4 Institute of Epidemiology, Disease Control and Research (IEDCR), Mohakhali-1212, Dhaka, Bangladesh; 5 Institute of Veterinary, Animal and Biomedical Sciences, Massey University, New Zealand; 6 Department of Livestock and Dairy Development, Government of Punjab, Punjab, Pakistan; 7 EcoHealth Alliance, New York, USA; 8 Department of Physiology, Biochemistry and Pharmacology, Faculty of Veterinary Medicine, Chittagong Veterinary and Animal Sciences University, Bangladesh

**Keywords:** bovine viral diarrhea virus, crossbred dairy cattle, enzyme-linked immunosorbent assay, seroprevalence, Bangladesh

## Abstract

**Aim:**

The study was conducted to determine the seroprevalence of bovine viral diarrhea virus (BVDV) and hematological features in crossbred dairy cattle in Chittagong, Bangladesh.

**Materials and Methods:**

The antibody against BVDV in crossbred dairy cattle serum was detected by indirect enzyme-linked immunosorbent assay. The association of different categorical variables in the prevalence of BVDV has been studied. Blood samples were collected and analyzed to know the hematological variations in the study population.

**Results:**

The overall seroprevalence of BVDV in the study area was 51.1% (95% confidence interval [CI], 40.5-61.5). Among different physiological stages of animals, the highest 57.1% (95% CI, 42.2-71.2) prevalence was in case of non-pregnant animals. Aborted cows were found to be significantly (p<0.05) more seropositive 77.8% (95% CI, 52.4-93.6) than the non-aborted cows (77.8%, 95% CI, 52.4-93.6, compared to 44.7%, 95% CI, 33.3-56.6, respectively). Cows having the history of retained placenta were found more positive than without the history of retained placenta (63.2%, 95% CI, 38.4-83.7, compared to 54.7%, 95% CI, 40.4-68.4, respectively). Among the animals of different age groups, BVDV seroprevalence was higher 61.3% (95% CI, 42.2-78.2) in animals of more than 3 years up to 5 years, whereas 32% was in case of 0-1-year-old. Significant variation found in different geographical areas of the study area. Hematological analyses have shown variation between the BVDV positive and negative animals.

**Conclusion:**

Seroprevalence of BVDV found to be high in the study area is also economically important and cause significant damage to the production industry. Therefore, it is necessary to conduct effective control measures to reduce the burden of BVDV.

## Introduction

Bovine viral diarrhea (BVD) is a disease of cattle, yaks, sheep, water buffalo, and even-toed ungulates [1-3] and is caused by a single-stranded positive-sense RNA virus of the genus Pestivirus. Genus Pestivirus is in the family Flaviviridae and includes four species - BVD virus 1 (BVDV 1), BVD virus 2 (BVDV 2), classical swine fever virus, and Border disease virus [4]. A new virus called HoBi-like BVDV 3 has recently been acknowledged in Thailand, Europe, and Brazil [5-8].

The disease has a global distribution and impacts animal health and reproductive performance, resulting significant financial losses [9]. Fever, leukopenia, diarrhea, decreased milk production, congenital defects, lameness, and reproductive problems such as early embryonic death, abortion, still birth, and mummification are the common manifestation in BVDV affected animals [10-14]. In some cases, animals do not exhibit clinical manifestation, but experience immunosuppression [15]. Animals diseased with noncytopathic BVDV during an early pregnancy period may yield persistently diseased animals and are responsible for viral shedding in the herd [16]. Thus, identification and elimination of such animals are important for the accomplishment of eradication programs [17].

Bangladesh has a cattle population of ~24 million animals, with 145 ruminants per square kilometer, representing one of the most densely livestock-populated countries in the world. The livestock sector contributes about 3% to the agricultural gross domestic product and facilitates about 15% of national employment [18]. Considering the potential financial impact of BVDV in a such large cattle population and the known presence of this disease in bordering India [2], very limited research has been carried out on the prevalence of this disease in Bangladesh [19]. Until recently, a study performed in three district veterinary hospitals including Chittagong during 2009-2010 confirmed the type of circulating BVDV in Bangladesh. Three percent of cattle sera samples were tested positive for BVDV confirmed by enzyme-linked immunosorbent assay (ELISA), while nucleotide sequences confirmed the rare HoBi-like pestivirus of BVDV-3 in the cattle population [18]. A limited study has been conducted to assess the burden and there thought to have significant knowledge gaps on true BVDV burden in Bangladesh. Therefore, the present research work was envisaged to determine the seroprevalence of BVDV in crossbred dairy cattle, a critical sector for economic and agricultural output in the country, and demonstrate their hematological features associated with infection.

## Materials and Methods

### Ethical approval

The research study was approved by the Ethics Committee of the Chittagong Veterinary and Animal Sciences University. Consent was attained from the owners of the cattle before collection of blood samples.

### Geography of the study area

**Figure-1:**
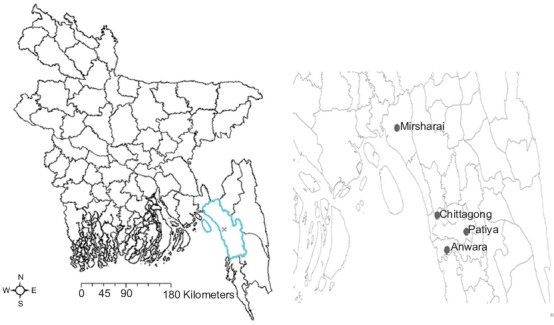
Geographical location of study area highlighted in Bangladesh map (right inset, larger view of the study area).

Chittagong is one of the important sea-belt zones in southeastern part of Bangladesh, surrounded on the north by Tripura State of India, on the east by hilly districts Khagrachhari, Rangamati and Bandarban, and Cox’s Bazar district on the south and the west by the Bay of Bengal. The total area of the district is 5282.92 km^2^ of which 1700 km^2^ includes coastal area. The district lies between 21°54’ and 22°59’ north latitude and between 91°17’ and 92°13’ east longitude (Figure-1). Sampling was conducted in the north, south, east, and central zones (Mirsharai, Anowara, Patiya, and Chittagong metropolitan area, respectively) of the district. The number of dairy farms documented in these zones ranges from 9 to 159 farms.

### Sample size determination

The required sample size was based on 3% prevalence (16/638) for BVDV in cattle admitted in Chittagong, Netrokona, and Dinajpur government veterinary hospitals in the 2009-2010 [18]. We assumed that cattle might show 6% BVDV seropositivity in the study area population. Based on this assumption, we estimated a sample size requirement of 86 cattle (population size 10,000, confidence limits 5%, design effect 1.0, and clusters 1). The sample size was calculated using the Epi InfoTM 7.1.5.0 software (Windows version).

### Sample collection

Blood samples were collected from dairy cattle (n=94) during July 2013 to April 2014. The cattle were selected randomly based on the interest of owners to test their animals for BVDV. The enrolled animals were apparently healthy and/or had a history of one or more select clinical indications (including abortion, retention of placenta, and diarrhea). Farm owners were been interviewed, and information on age, sex, body condition scored (BCS), parity, breed, clinical signs, and pregnancy status of animals were recorded. BCS was scored in a scale of 1-5 which characterized worst to best physical condition based on bony prominence and deposition of subcutaneous fat [20]. A research team, comprised two experienced veterinary doctors and a veterinary assistant visited the farms, and ~6-8 mL blood were aseptically collected from the jugular vein in sterile vacutainers using separate needles. Following collection, 2 mL of blood with EDTA anticoagulant was stored on ice and transported to the Department of Physiology, Biochemistry and Pharmacology (DPBP) Laboratory of Chittagong Veterinary and Animal Sciences University (CVASU) for hematological examination. The remaining blood samples were left to clot for 60-120 min at room temperature, and serum was obtained by centrifugation at 3000 rpm for 10 min. Serum samples were then stored at −20°C and analyzed at the Poultry Research and Training Centre Laboratory of CVASU.

### Laboratory investigation

Serum was screened with an indirect ELISA kit for detection of BVDV antibody (SVANOVIR^®^ BVDV-Ab, Svanova Biotech AB, Uppsala, Sweden) as per the manufacturer’s instructions. The optical density was measured using a spectrophotometer (Mindray MR-96A) at a single wavelength of 450 nm. The sensitivity and specificity of the test were 100% and 98.2%, respectively [21].

Blood with EDTA was examined for total erythrocyte count (TEC), hemoglobin (Hb), packed cell volume (PCV), and total leukocyte counts (TLC). Differential leukocyte counts (DLC) were estimated on Wright-stained blood smears [22].

### Data analysis

All obtained field data and laboratory results were stored and cleaned in Excel Spreadsheet (Microsoft Office, 2013). Statistical analysis was performed in STATA13 (STATA Corp, USA). Hematological parameters (TEC, TLC, PCV, Hb, lymphocyte, monocyte, neutrophil, eosinophil, and basophil) between the BVDV positive and negative animals were compared by t-test for statistical significance. The Pearson Chi-square test was used to assess the seroprevalence of BVDV. A p≤0.05 was considered statistically significant.

## Results

### Seroprevalence of BVDV

Of the 94 cattle tested, 51.1% (95% confidence interval [CI], 40.5-61.5) (n=48) were found to be seropositive for BVDV (Table 1). Although the number of enrolled animals varies geographically, higher seroprevalence has been observed in the north zone of the study area (82.8%, 95% CI, 64.2-94.2). Cattle within age range 3-5 years and >5 years of age represent higher seropositivity than other age groups (Table 1). Although seropositivity was proportionately higher in non-pregnant cows 57.1% (95% CI, 42.2-71.2), there was no significant relationship based on pregnancy condition of the enrolled cows. A significant relationship was found among aborted and non-aborted cows. In case of aborted cows, the prevalence of BVDV was 77.8% (95% CI, 52.4-93.6) in comparison to 44.7% (95% CI, 33.3-56.6) of the non-aborted cows. Prevalence of BVDV was higher in the cows having a previous history of retention of placenta than that of the cows having no such clinical record. In case of the former group, i.e., cows with retained placenta, the prevalence was 63.2% (95% CI, 38.4-83.7), whereas among the non-retained placenta cows the rate was 54.7% (95% CI, 40.4-68.4). In the study, among 41 animals with reported episodes of diarrhea in the last 1 year, 43.9% (95% CI, 28.5-60.3) animals were found seropositive to BVDV, though 30 non-diarrheic animals were positive to BVDV, with seroprevalence of 56.6% (95% CI, 42.3-70.2). BVDV was highest 63.6% (95% CI, 40.7-82.8) in those animals having BCS 2 and lowest 38.5% (95% CI, 13.9-68.4) in animals with BCS 3.5. Cows having high parity were found to be more affected with BVDV in relation to the lower number of parities, with the exception of cows having 9 parities showing a lower prevalence rate. In that case, the lowest 41.2% (95% CI, 18.4-67.1) seroprevalence was found in cows with single parity, whereas highest has been found in cows having 6-7 parities.

**Table-1:** Association of different categorical variables in the prevalence of BVDV in cattle.

Variable	Category (N)	No. positive	% Prevalence (95% CI)	p value
Zone distribution	South (54)	16	29.6 (18.0-43.9)	0.00
	Central (11)	8	72.7 (39.0-94.0)	
	North (29)	24	82.8 (64.2-94.2)	
Age (years)	0-1 (22)	7	31.8 (13.9-54.9)	0.15
	>1-3 (21)	10	47.6 (25.7-70.2)	
	>3-5 (31)	19	61.3 (42.2-78.2)	
	>5 (20)	12	60.0 (36.1-80.9)	
Pregnancy status	Pregnant (29)	13	44.8 (26.4-64.3)	0.33
	Non-pregnant (49)	28	57.1 (42.2-71.2)	
Abortion status	Aborted (18)	14	77.8 (52.4-93.6)	0.04
	Non-aborted (76)	34	44.7 (33.3-56.6)	
Retained placenta	Yes (19)	12	63.2 (38.4-83.7)	0.52
	No (53)	29	54.7 (40.4-68.4)	
Diarrhea	Yes (41)	18	43.9 (28.5-60.3)	0.31
	No (53)	30	56.6 (42.3-70.2)	
BCS	2 (22)	14	63.6 (40.7-82.8)	0.5
	2.5 (12)	6	50.0 (21.1-78.9)	
	3 (47)	23	48.9 (34.1-63.9)	
	3.5 (13)	5	38.5 (13.9-68.4)	
Parity status	1 (17)	7	41.2 (18.4-67.1)	0.37
	2 (12)	8	66.7 (34.9-90.1)	
	3 (25)	15	60.0 (38.7-78.9)	
	4 (7)	5	71.4 (29.0-96.3)	
	6 (3)	3	100.0 (29.2-100*)	
	7 (1)	1	100.0 (2.5-100*)	
	9 (2)	1	50.0 (1.3-98.7)	
Total	(94)	48	51.1 (40.5-61.5)		

N=Number of animals;

* one-sided, 97.5% CI; BCS-2, cachectic (severe), protruding rib with prominent pin bone; BCS-2.5, Cachectic, protruding rib with prominent pin bone; BCS-3, rough body condition and ribs are moderately visible; BCS-3.5, good body condition; parity status, the number of times cow has given birth. BCS=Body condition score, CI=Confidence interval, BVDV=Bovine viral diarrhea virus

### Hematological features among seropositive and negative animals

None of the values showed the significant variation in hematological values of BVDV positive and negative animals. In BVDV positive animals, TEC was 6.18±0.13 (×106/cumm), whereas in BVDV negative animals the value was 6.30±0.13 (×106/cumm). TLC value of BVDV positive and negative animals were 8.61±0.35 and 9.23±0.44 (×103/cumm). The PCV was 28.48±0.61 and 27.91±0.68 (%), respectively. Hb percentage was 8.65±0.14 and 8.57±0.14 (%) in case of BVDV positive and negative animals (Table 2).

**Table-2:** Comparative study of hematological parameters among BVDV positive and negative animals

Variables	BVDV serodiagnosis	Mean±standard error	95% Confidence interval	p value
TEC (×10^6^/cumm)	Positive	6.18±0.13	5.91-6.45	0.50
	Negative	6.30±0.13	6.03-6.45	
TLC (×10^3^/cumm)	Positive	8.61±0.35	7.91-9.31	0.27
	Negative	9.23±0.44	8.34-10.12	
PCV (%)	Positive	28.48±0.61	27.25-29.71	0.54
	Negative	27.91±0.68	26.55-29.27	
Hb (g/dl)	Positive	8.65±0.14	8.38-8.93	0.69
	Negative	8.57±0.14	8.29-8.86	

TEC=Total erythrocyte count, Hb=Hemoglobin, PCV=Packed cell volume, TLC=Total leukocyte counts, BVDV=Bovine viral diarrhea virus

In case of DLC, the only significant difference was found in basophil although others did not show significant variation. Lymphocyte was found to be 63.63±1.47% and 65.74±1.81%, whereas monocyte was found to be 5.34±0.53% and 5.28±0.55%, for BVDV positive and negative animals, respectively. In case of BVDV seropositive animals, the neutrophil was 20.96±1.15%, compared to 18.54±1.54% in the case of seronegative animals. Eosinophil was 9.38±0.75% and 9.33±0.86%, respectively, in BVDV positive and negative animals. The basophil was 0.69±0.13% and 1.13±0.14%, respectively, in case of BVDV positive and negative animals (Table 3).

**Table-3:** Distribution of different types of leukocytes in BVDV seropositive and negative animals.

Variables	BVDV serodiagnosis	Mean±standard error	95% confidence interval	p value
Lymphocyte (%)	Positive	63.63±1.47	60.67-66.58	0.36
	Negative	65.74±1.81	62.10-69.38	
Monocyte (%)	Positive	5.34±0.53	4.29-6.41	0.93
	Negative	5.28±0.55	4.18-6.38	
Neutrophil (%)	Positive	20.96±1.15	18.65-23.27	0.21
	Negative	18.54±1.54	15.43-21.65	
Eosinophil (%)	Positive	9.38±0.75	7.87-11.05	0.97
	Negative	9.33±0.86	7.60-10.88	
Basophil (%)	Positive	0.69±0.13	0.43-0.95	0.02
	Negative	1.13±0.14	0.85-1.41	

BVDV=Bovine viral diarrhea virus

## Discussion

Forty-eight (51.1%, 95% CI, 40.5-61.5) of the 94 cattle were positive for BVDV antibody (Table 1). This finding is consistent with findings from prior serological studies on BVDV. In Iran, these indicated seroprevalence of 51.75% in Ahvaz and 51.58% in Tehran [23] and a similar observation was recorded in north Italy [24] and slightly higher in Turkey (60%) [25]. The overall seroprevalence of more than 50% recorded in the study animals is also in agreement with previously reported prevalence (50-90%) in various studies in USA, Canada, Germany, England, Kenya, New Zealand, Australia, Colombia, and Argentina [26-29]. Nevertheless, the seroprevalence slightly varies with the findings (70-100%) in various studies worldwide [28-31]. This variation in seroprevalence in different countries may be due to differences in cattle population age, cattle density, herd size, housing systems, biosecurity, and management practices, which in general could be important risk factors in transmission and persistence of BVDV [5,32]. The high seroprevalence demonstrates that the BVDV situation is critical in crossbred dairy cattle in the selected geographical zones in Chittagong. In the current study, variation was found in the seroprevalence of BVDV in the south, central, and north zones of Chittagong. The study revealed that among the three zones the seroprevalence was highest in the north, comprising 82.8% of animals sampled (95% CI, 64.2-94.2), whereas the south was lowest with 29.6% seropositivity (95% CI, 18.0-43.9). The difference was statistically significant (p≤0.05). Lee *et al*. [33] also mentioned finding variations in the prevalence of BVDV in dairy cows of different locations in South Korea; the range of results of our present study differed more than Lee *et al.*, which may be due to fewer herds and samples in our study. The variation may also be due to the geographical variation, management and husbandry practices between the two countries. The presence of a high number of seropositive animals was an indirect indication of the presence of one or more persistently infected animals in the tested herds.

Seroprevalence varied among different age groups; the highest 61.3% (95% CI, 42.2-78.2) was found in >3-5 years age groups and lowest 31.8% (95% CI, 13.9-54.9) in the age group 0-1 year. There was no significant (p>0.01) difference in the seroprevalence of BVDV in different age groups of animals, but the results from our study demonstrated that the number of seropositive animals’ increases with age. In the 1-3 and consecutive years of life, the number of seropositive animals increased reaching the peak in a group of animals aged >3-5 and >5 years (61% and 60% of seropositive animals, respectively). Animal age was in direct correlation with the number of seropositive animals.

BVDV seropositivity based on pregnancy status of cows showed no significant association, though there was proportionate variation among pregnant and non-pregnant cows (Table 1). This was slightly lower than indicated by Wittum *et al*. [32] who found 50% seropositive calves in a suspected BVDV-infected herd in Alabama, USA. In Lithuania, Mockeliuniene *et al*. [34] observed that 57.6% of cows were seropositive, which is very similar 57.1% (95% CI, 42.2-71.2) to our present study. There was a high prevalence of BVDV in cattle having the previous history of abortion. In this regard, 18 out of 94 animals had abortion history, with 77.8% (95% CI, 52.4-93.6) BVDV positive (Table 1), this indicates that, there was a significant difference (p=0.03) between two groups of cows. In the case of aborted fetus in Iran, 17.90% and 18.49% seroprevalence was detected by antigen captured ELISA [23,35]. This high variation in the prevalence of BVDV in between the sera samples and aborted fetus may be due to the difference in the samples or the abortion may be caused by other causal agents. On the other hand, Ahmad *et al*. [13] reported 78.57% aborted animals were seropositive at an age level 24-36 months in Canada by virus neutralization test (VNT) in Holstein cattle, which is very close to our present study. In a research work, Rezaeisaber *et al*. [36] demonstrated 44% aborted cows were infected to BVDV in Iran, which is highly deviated from our study result. Yildirim *et al*. [37] carried out a research on investigation of a possible involvement of BVDV in abortion of dairy cattle in Turkey. They observed 52.9% aborted cows as the seropositive by the VNT. The presence of retained placenta was investigated as a complementary factor, which can have an influence on the prevalence of BVDV infection. The cows having a history of retained placenta showed 63.2% (95% CI, 38.4-83.7) seropositivity to our test, whereas, 54.7% (95% CI, 40.4-68.4) cows without a history of BVDV showed positivity. Diarrhea has negative correlation on the prevalence of BVDV in the study animals. In case of diarrheic animals, the rate of seropositivity was 43.9% (95% CI, 28.5-60.3), where in case of non-diarrheic animals the rate was 56.6% (95% CI, 42.3-70.2). It might be due to the other causal agents of diarrhea in the cattle such as bacterial, parasitic or any other viral agents except BVDV. Kabongo and Van Vuuren [38] in his research in South Africa found 20% and 13.3% cattle with BVDV having diarrhea along with pyrexia and only diarrhea, respectively. There is a great difference between our present study and his study. This deviation probably due to the presence of other causal agents of diarrhea. BCS revealed a negative correlation in the prevalence of BVDV. In our present study, it was found that animals with low BCS is prone to be mostly infected with the BVDV, whereas, animals with high BCS were found to be less infected with BVDV. Percentage of BVDV gradually increased with the decrease of BCS. In the present study, it indicates that animals with BCS 2 were highly infected with BVDV 63.6% (95% CI, 40.7-82.8), whereas, animals with BCS 3.5 were less 38.5 (13.9-68.4) infected with the BVDV. The high rate in low BCS animals may be due to the low immunity level of the body.

The study evidenced a positive correlation between the 5^th^ and 6^th^ parity of cows and the seropositivity of BVDV. A high percentage of seropositive cattle are directly related to the number of parity of cows. Lee *et al*. [33] detected 71.9% positive cases in those cows with more than 4 parities and 48.9% cases in cows with single parity, which is slightly higher than our present study.

In this study, hematological tests demonstrated TEC value 6.18±0.13 million/cumm in BVDV positive cattle and in case of negative it was 6.30±0.13 million/cumm. It is nearly similar to the finding of the previous research of Alsaad *et al*. [39], where the authors mentioned 6.71±1.76 and 6.92±1.34 million/cumm in positive and negative cattle, respectively. TLC was found 8.61±0.35 and 9.23±0.44 thousand/cumm in BVDV positive and negative cattle, respectively, in this study, whereas, in Iraqi calves, it was found 10.41±1.52 and 12.37±1.54 thousand/cumm in positive and negative animals [39]. 28.48±0.61% and 27.913±0.68% PCV was detected in our study from BVDV positive and negative animals. In local Iraqi calves it was found 47.00±4.25 and 33.32±2.41%, respectively, in BVDV positive and control animals. Genital infection has a negative impact in the PCV of the cattle, and 31.8±1.58% PCV was detected in cows with genital infection [39,40].

In this study, the Hb value was detected as 8.65±0.13 and 8.57±0.14 g/dl in BVDV positive and negative animals, which is opposite to the results described in the previous work in Iraq [39]. The variation between the result of this study and of these in may be due to the age difference. In Iraq, the study was performed in calves, but our study was conducted in different age groups, where most of the cattle were above 2-year-old, and Hb is found to be decreased with the increment of age. Ruginosu *et al*. [40] mentioned 9.20±0.30 g/dl Hb in cows with genital infection, which reflects that due to genital infection Hb value decreased. Our result may be decreased due to the presence history of abortion and retained placenta.

Lymphocyte was detected 63.625±1.47% and 65.74±1.81% in case of BVDV positive and negative animals, respectively. This value is very higher than the research work of Iran, where lymphocyte was found as 40.84±2.54 and 47.52±3.61% in seropositive and negative animals, respectively. According to both study, we can claim that cows with BVDV positivity contain less number of lymphocyte than the control or negative animals. In the case of monocyte, it is reverse, where our study detected 5.34±0.58 and 5.28±0.57% in positive and negative animals, respectively, but it was 4.83±2.23 and 4.53±1.11% in case of positive and negative animals, respectively, according to Alsaad *et al*. [39]. The values obtained in case of cattle having the genital and puerperal infection was 1.70±0.20, which is lower than our present study [40]. Neutrophil in our study was very much lower than the previous related study such as 48.62±4.32 and 39.50±0.67% in case of BVDV affected and genitally affected animals, respectively [39,40]. The reason of the variation may be due to breed, stage of infection, immunity and susceptibility of the host. On the other hand, eosinophil was higher in this study (9.38±0.75%) than the previous study (4.71±1.34%) conducted by Alsaad *et al.*, 2012. Basophil, another component of blood was detected 0.69±0.13% and 1.13±0.14% in BVDV positive and negative cases, respectively. In our study in case of positive case, the value was lower than the negative case, whereas in the previous study it was reverse such as 1.40±0.12% and 1.30±0.25% in case of positive and negative cases, respectively [39].

There are some limitations and further directions of this study. The sample only represents a specific region of the country and did not have complete coverage of farms in the region due to staffing and budget limitations. However, the findings indicate the first detection of BVDV in apparently healthy cattle in the Chittagong region. To gain more precise understanding of seroprevalence in Bangladesh, a nationally-representative study of dairy cattle is needed.

## Conclusion

In addition to America, Europe, Africa, Australia, New Zealand, the circulation of BVDV in crossbred cattle in South-East Asia, including Bangladesh (a subtropical country), draws insights into the geographical spread and probable emergence of this zoonotic virus in many other countries. The very limited study of this disease in Bangladesh on BVDV presents a unique opportunity for future expansion of our study to demonstrate the seroprevalence and hematological features of BVDV in crossbred commercial dairy cattle in other regions of Bangladesh. The research findings would convey home-messages to the associated stakeholders that could improve the health and productivity of animals in a subtropical country like Bangladesh. Given the demonstrated economic impact of BVDV in other countries, the study of the risk factors present in Bangladesh is also warranted to mitigate its potential impact.

## Authors’ Contributions

MAU: Conception and design, sample collection, laboratory works, data analysis and interpretation, and review of the manuscript. ASMLA: Supervision, conception and design, drafting manuscript, review of analysis, interpretation and critical review of the manuscript. KI: Conceptualization, writing and drafting the manuscript, assist in laboratory works, statistical analysis and interpretation of the data, GPS mapping, critical review of the manuscript. MZI: Conception and design, statistical analysis, interpretation, critical review of the manuscript. AM: Analysis overview, critical review of the manuscript. AI: Build map as per GPS coordinate. KMFI: Reference list arrangement, cross-checking and critical review of the manuscript. AA: Overall supervision, conception and design, instruction and review of analysis, interpretation, and critical review of the manuscript. All authors read and approved the final manuscript.
